# *amoA*-based consensus phylogeny of ammonia-oxidizing archaea and deep sequencing of *amoA* genes from soils of four different geographic regions

**DOI:** 10.1111/j.1462-2920.2011.02666.x

**Published:** 2012-02

**Authors:** Michael Pester, Thomas Rattei, Stefan Flechl, Alexander Gröngröft, Andreas Richter, Jörg Overmann, Barbara Reinhold-Hurek, Alexander Loy, Michael Wagner

**Affiliations:** 1 Department of Microbial EcologyAlthanstrasse 14, A-1090 Vienna, Austria; 2 Department of Computational Systems BiologyAlthanstrasse 14, A-1090 Vienna, Austria; 3 Department of Chemical Ecology and Ecosystem Research, University of ViennaAlthanstrasse 14, A-1090 Vienna, Austria; 4 Institute of Soil Science, University of HamburgAllende-Platz 2, D-20146 Hamburg, Germany; 5 Leibniz-Institut DSMZ-Deutsche Sammlung von Mikroorganismen und ZellkulturenInhoffenstraße 7B, D-38124 Braunschweig, Germany; 6 Department of Microbe-Plant Interactions, University of BremenPostfach 330440, D-28334 Bremen, Germany

## Abstract

Ammonia-oxidizing archaea (AOA) play an important role in nitrification and many studies exploit their *amoA* genes as marker for their diversity and abundance. We present an archaeal *amoA* consensus phylogeny based on all publicly available sequences (status June 2010) and provide evidence for the diversification of AOA into four previously recognized clusters and one newly identified major cluster. These clusters, for which we suggest a new nomenclature, harboured 83 AOA species-level OTU (using an inferred species threshold of 85% *amoA* identity). 454 pyrosequencing of *amoA* amplicons from 16 soils sampled in Austria, Costa Rica, Greenland and Namibia revealed that only 2% of retrieved sequences had no database representative on the species-level and represented 30–37 additional species-level OTUs. With the exception of an acidic soil from which mostly *amoA* amplicons of the *Nitrosotalea* cluster were retrieved, all soils were dominated by *amoA* amplicons from the *Nitrososphaera* cluster (also called group I.1b), indicating that the previously reported AOA from the *Nitrosopumilus* cluster (also called group I.1a) are absent or represent minor populations in soils. AOA richness estimates on the species level ranged from 8–83 co-existing AOAs per soil. Presence/absence of *amoA* OTUs (97% identity level) correlated with geographic location, indicating that besides contemporary environmental conditions also dispersal limitation across different continents and/or historical environmental conditions might influence AOA biogeography in soils.

## Introduction

Ammonia oxidation to nitrite is the rate limiting step in nitrification and as such an important part of the global biogeochemical nitrogen cycle. For more than hundred years, it has been known that this process can be performed by chemolithoautotrophic bacteria (Winogradsky, 1890) and detailed phylogenetic analyses showed that all recognized ammonia oxidizing bacteria (AOB) are confined to two phylogenetic lineages within the *Gamma*- and *Betaproteobacteria* (Teske *et al*., 1994; Purkhold *et al*., 2000). The recent discovery of ammonia-oxidizing archaea (AOA) revealed that an additional group of microorganisms is able to catalyse this process (Venter *et al*., 2004; Könneke *et al*., 2005; Treusch *et al*., 2005). Classified initially by 16S rRNA phylogeny as *Crenarchaeota* (Delong, 1992; Fuhrman *et al*., 1992), recent analyses based on comparative genomics and phylogeny of concatenated genes placed these microorganisms into the new archaeal phylum *Thaumarchaeota* (Brochier-Armanet *et al*., 2008; Spang *et al*., 2010; Pester *et al*., 2011). Although being members of two different domains of life, AOB and AOA exploit homologous ammonia monooxygenases, that are members of the copper-containing membrane-bound monooxygenase (CuMMOs) enzyme family (Tavormina *et al*., 2011) in order to activate ammonia and thus both groups carry *amo*-genes in their genomes.

In the mainly negatively charged soil matrix, nitrification increases the mobility of inorganic nitrogen by converting the positively charged ammonium to the negatively charged nitrate. When overstimulated by heavy Nfertilization in agricultural settings, nitrification thus leads to soil acidification, increased production of the greenhouse gas nitrous oxide, and increased N-loss due to leaching of the produced nitrate from soil and subsequent pollution of streams and groundwaters (Stevenson, 1986). In many soils, archaeal *amoA* genes (coding for the alpha-subunit of the ammonia monooxygenase) outnumber their bacterial counterparts with both, archaeal and bacterial *amoA* genes being transcribed (Leininger *et al*., 2006). Generally, AOA seem to dominate ammonia oxidation in soil under low nitrogen availability (< 15 µg NH_4_^+^-N per g dw soil), whereas AOB become more competitive at higher nitrogen loads (Erguder *et al*., 2009; Jia and Conrad, 2009; Di *et al*., 2010; Gubry-Rangin *et al*., 2010; Zhang *et al*., 2010; Pratscher *et al*., 2011; Verhamme *et al*., 2011; Xia *et al*., 2011). A preference of AOA for low substrate concentrations is consistent with the physiological characterization of the marine AOA *Candidatus* Nitrosopumilus maritimus, which has a substrate threshold for total ammonium (NH_4_^+^ + NH_3_) as little as 10 nM and thus by far outcompetes known AOB under low ammonia concentrations (Martens-Habbena *et al*., 2009). In addition, *Candidatus* Nitrososphaera gargensis, a moderate thermophile closely related to AOA typically found in soils, has been shown to be inhibited by total ammonium concentrations in the lower mM-range (Hatzenpichler *et al*., 2008) giving further physiological support for the observed preference of AOA for low electron donor concentrations. However, the soil AOA *Candidatus* Nitrosotalea devanaterra and *Candidatus* Nitrososphaera viennensis grow in media containing total ammonium concentrations as high as 10 and 15 mM respectively (Lehtovirta-Morley *et al*., 2011; Tourna *et al*., 2011), and archaeal *amoA* transcripts were detected in soils amended with 10 mM total ammonium (Treusch *et al*., 2005), indicating that AOA exist that are adapted to higher nitrogen loads.

The ability of AOA to be active under a wide range of total ammonium concentrations goes along with the detection of archaeal *amoA* genes in soils ranging from acidic to alkaline pH (Erguder *et al*., 2009). Soil pH has a direct effect on the availability of ammonia (pK_a_ of NH_3_ : NH_4_^+^ = 9.25), which is currently believed to be the substrate of the ammonia monooxygenase (Suzuki *et al*., 1974; Frijlink *et al*., 1992). However, this assumption is based solely on studies using the AOB *Nitrosomonas europaea* as model organism and the recent cultivation and physiological characterization of the first acidophilic ammonia oxidizer *Candidatus* N. devanaterra indicated that soil AOA might have developed new mechanisms of ammonia oxidation under acidic conditions or low ammonia availability (Lehtovirta-Morley *et al*., 2011).

Another factor that may influence AOA activity is organic carbon, which inhibits in low concentrations the growth of *Candidatus* N. maritimus and of the thermophile *Candidatus* Nitrosocaldus yellowstonii (Könneke *et al*., 2005; de la Torre *et al*., 2008). However, organic carbon (as pyruvate) is essential for high growth yields of the soil AOA *Candidatus* N. viennensis (Tourna *et al*., 2011). Furthermore, in soils the nature of the ammonia source might be of relevance. AOA activity was detected when ammonia was supplied as mineralized organic N derived from composted manure or soil organic matter while AOB-dominated activity was measured with ammonia originating from inorganic fertilizer (reviewed in Schleper and Nicol, 2010). In addition, (meta-)genome analyses (Hallam *et al*., 2006; Martin-Cuadrado *et al*., 2008; Walker *et al*., 2010) and environmental studies (Ouverney and Fuhrman, 2000; Herndl *et al*., 2005; Ingalls *et al*., 2006) indicate that AOA might be able to switch from autotrophic ammonia oxidation to a mixotrophic and possibly even heterotrophic lifestyle, a capacity that may contribute to their numerical dominance in soils.

Most studies targeted at characterizing and quantifying AOA in soil use the functional and phylogenetic marker gene *amoA*. However, no recent and encompassing phylogenetic analysis of all the environmentally retrieved *amoA* sequences, which could be used as basis for such studies, is available. Thus, the different publications in this rapidly growing field contain *amoA* trees inferred from different data sets and treeing methods (often only neighbour joining trees are presented) and use different terminologies for different AOA lineages complicating comparisons and meta-analyses. In this study, we established a curated reference database of all publicly available archaeal *amoA* sequences and calculated a consensus tree that integrates over different phylogeny inference methods and displays unresolved diversification with multifurcations (Ludwig *et al*., 1998). Based on these analyses, we examined AOA in a selection of 16 geographically and climatically very distinct soils by *amoA* pyrosequencing using Roche's GS FLX Titanium 454 platform in an effort to estimate their overall diversity in these systems and to elucidate how representative the established reference data set is.

## Results

### Archaeal *amoA* diversifies into five major clusters

**Fig. 1:**
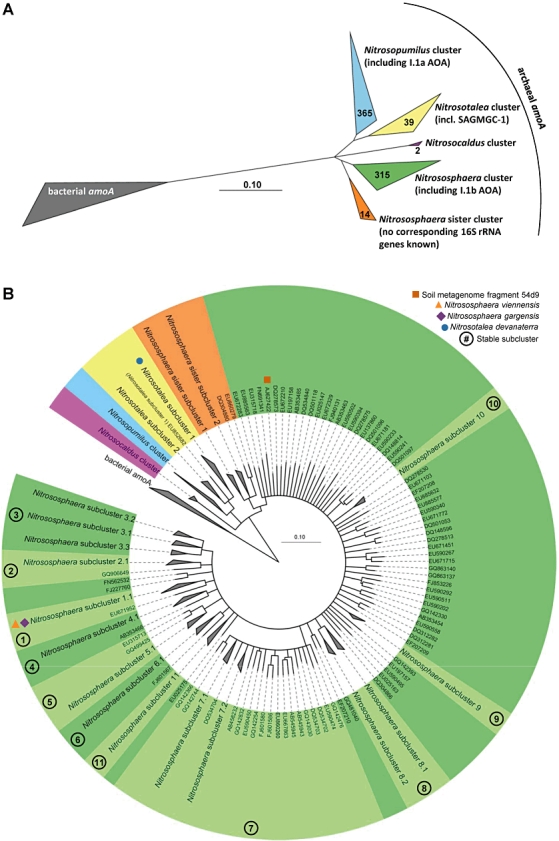
Consensus tree illustrating the five major clusters of archaeal *amoA* (designations in brackets refer to frequently used corresponding cluster names in AOA 16S rRNA trees) (A) and the diversification of the *Nitrososphaera*, *Nitrososphaera* sister, and *Nitrosotalea* cluster at the second and third phylogenetic level (B). The tree was determined using 592 unambiguously aligned positions of a data set of 735 representing nucleic acid *amoA* sequences. Each reference sequence is representative for a group of *amoA* sequences with an identity of ≥ 97%. For the radial overview tree, numbers within major lineages represent numbers of representing sequences (A). For the detailed circular tree, numbers in circles represent the second phylogenetic level (e.g. *Nitrososphaera* subcluster 1), whereas the third phylogenetic level is directly indicated at the tree branch (e.g. *Nitrososphaera* subcluster 1.1); sequences that did not form stable subclusters of more than three representatives kept the phylogenetic affiliation of the higher phylogenetic level and are indicated by their NCBI accession number (B). A corresponding detailed circular tree of the *Nitrosopumilus* cluster is given in Fig. S1. The consensus tree and the source alignment of representing sequences can be found in File S1. The scale bar indicates 10% estimated sequence divergence based on a Jukes-Cantor corrected distance matrix analysis.

To perform a comprehensive analysis of archaeal *amoA* phylogeny, we screened the NCBI (Benson *et al*., 2011), IMG/M (Markowitz *et al*., 2008) and Camera (Sun *et al*., 2011) databases for all publicly available entries of this gene. This resulted in retrieval of 12 356 sequences, which were grouped into 735 clusters of ≥ 97% sequence identity. For each of these clusters a representing sequence with a length of ≥ 592 bp was selected, which eliminated sequence redundancy and reduced the amount of sequences to a manageable amount for the calculation-intensive phylogeny inference methods. Together, these representing sequences evenly covered the known sequence space of archaeal *amoA*. Based on this database, we constructed distance matrix, maximum parsimony, and maximum likelihood trees, which were combined in a consensus tree using the majority rule that defines that a cluster must be represented in at least two of the three different trees. Expanding on previous less comprehensive analyses (e.g. Francis *et al*., 2005; Prosser and Nicol, 2008), our consensus archaeal *amoA* tree is composed of five major monophyletic clusters, which we named, if possible, after the genus name of the first cultured representative of each cluster (Fig. 1A). The *Nitrosopumilis* cluster (previously also referred to as marine or I.1a AOA lineage especially in 16S rRNA based trees, DeLong, 1998) contained 365 representing sequences, while 315 representing sequences were assigned to the *Nitrososphaera* cluster (previously also referred to as soil or I.1b AOA lineage, DeLong, 1998), demonstrating that both clades encompass highly diverse groups of microorganisms. The *Nitrosocaldus* (previously also called ThAOA or HWCGIII lineage, de la Torre *et al*., 2008; Prosser and Nicol, 2008), *Nitrosotalea* (also referred to as group I.1a associated, Lehtovirta-Morley *et al*., 2011), and *Nitrososphaera* sister cluster (previously not recognized) are less diverse based on the current databases and contain 2, 39 and 14 representing sequences respectively.

The *Nitrosotalea* cluster forms a monophyletic group with the *Nitrosopumilus* cluster as outlined previously (Lehtovirta-Morley *et al*., 2011). Similarly, the newly recognized *Nitrososphaera* sister cluster, shared a common ancestor with the *Nitrososphaera* cluster to the exclusion of all other AOA clusters, but so far contains no cultured representatives or metagenome sequences with a corresponding 16S rRNA gene or other phylogenetic marker. In both cases, representing sequences between the respective sister clusters were at maximum 84% identical, whereas highest pairwise identity of representing sequences between all other clusters was lower ranging from 74–79% (Table S1).

The five major *amoA* clusters (first phylogenetic level) were hierarchically subdivided into a second (e.g. *Nitrososphaera* subcluster 1) and third (e.g. *Nitrososphaera* subcluster 1.1) phylogenetic level, guided by the branching order in the tree, i.e., by following the first and second (multi-)furcation within the cluster (Fig. 1B, Fig. S1). Those sequences that did not form stable subclusters of more than three representing sequences kept the cluster affiliation of the higher phylogenetic level (e.g. soil metagenome fragment 54d9 was only affiliated to the general *Nitrososphaera* cluster). Of all subclusters (phylogenetic level 2) within the *Nitrososphaera*, *Nitrososphaera* sister, *Nitrosotalea* and *Nitrosopumilus* cluster, none contained exclusively *amoA* sequences retrieved from a single environment like soil or ocean water, even if only the representing sequences were analysed. Subclusters within the *Nitrososphaera*, *Nitrososphaera* sister and *Nitrosotalea* cluster did not only contain sequences from terrestrial environments, but were intermixed with sequences from freshwater, wastewater, marine waters, estuary sediments and hot springs. The sequences assigned to the various *Nitrosopumilus* subclusters were often retrieved from aquatic environment but also here subclusters consisted of representing sequences originating from at least two of the following environments: marine water, hydrothermal vents, sponge symbionts, estuaries, wastewater, freshwater and soils (for details please refer to the generated archaeal *amoA* ARB database in File S1).

**Fig. 2:**
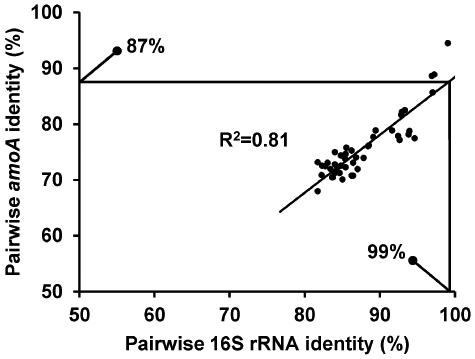
Pairwise comparison of 16S rRNA gene and archaeal *amoA* identities of all metagenome fragments and *Candidatus* species from which both genes are known. Sequences with less than 99% identity at the 16S rRNA gene level are considered to belong to different species (Stackebrandt and Ebers, 2006).

To estimate how many AOA species are currently known based on the analysed *amoA* data set, we performed a pairwise comparison of 16S rRNA gene and *amoA* identities of all metagenome fragments and archaeal *Candidatus* species from which both genes are known. Taking 99% sequence identity at the 16S rRNA gene level as an approximate threshold below which microbes can be assigned to different species (Stackebrandt and Ebers, 2006), we determined that *amoA* sequences with less than 87% nucleic acid sequence identity are likely to represent two different AOA species (Fig. 2). As this threshold value might still slightly change with the addition of sequence data from new AOA, we selected a more conservative value of 85% *amoA* sequence identity as a species threshold in our analyses, which we also recommend as threshold for future studies. Applying this threshold to the analysed *amoA* data set revealed that it represents 83 species-level operational taxonomic units (OTU), with most of these OTUs being present in the *Nitrosopumilus* (41 species-level OTUs) and *Nitrososphaera* (34 species-level OTUs) cluster followed by the *Nitrosotalea*, *Nitrososphaera* sister and *Nitrosocaldus* cluster (5, 2 and 1 species-level OTUs respectively). If the analysed *amoA* data set was extended to short *amoA* sequences (< 592 bp), which could not be used for phylogenetic analysis, the minimum number of currently known AOA increases to 108 species-level OTUs in total (data not shown).

### Considerable AOA species richness differences in a worldwide selection of soils

In order to assess and compare AOA diversity in a selection of geographically and climatically distinct soils from Southern Africa (Namibia), Central America (Costa Rica), Central Europe (Austria) and the arctic region (Greenland) (Table S2), a 454-amplicon sequencing approach of archaeal *amoA* was used. Replicate *amoA* amplicons from independent DNA extractions from each soil were pooled before sequencing to diminish within soil-heterogeneity and subjected to sequencing from the forward as well as the reverse end. To discriminate against sequencing errors, high-quality sequences were initially clustered on a 97% identity level (Kunin *et al*., 2009). Cluster representatives were further screened for sequencing errors using a frame shift detection procedure developed within this study (for details see *Experimental procedures*). This approach proved to be a powerful tool to identify and mask pyrosequencing errors of functional gene amplicons with about 45% of the obtained sequences being affected (data not shown). Automated chimera detection using the programs chimera slayer, pintail and bellerophon implemented in the Mothur software package (Schloss *et al*., 2009) or their stand-alone versions resulted always in a high rate of false positives as revealed by pairwise alignment of putative chimeras with their next relatives (data not shown). A possible explanation for this bad performance might be the insufficient sequence length of 454 reads or the malfunctioning of chimera detection programs, which were initially developed for the 16S rRNA gene but were here applied to protein coding *amoA* sequences. Therefore, chimera-detection was done manually by screening for massive misalignment of representing sequences at the 97% identity level, which resulted in 27 detected chimeras among 3835 representing sequences (detailed in *Experimental procedures*). Thereafter, *amoA* sequences were grouped according to their sequencing direction (forward or reverse) and analysed in parallel. Comparison of the forward and reverse sequenced data sets for all samples revealed no major skewing of species-level OTU abundances (Table S3).

In total, 110 059 high quality sequences with an average sequence length of 411 bp (range 250–509 bp) and an average of 6 879 sequences per soil were obtained (Table S3). Rarefaction analysis revealed that at the species level (85% *amoA* identity) all soils were sampled almost to saturation either in the forward, reverse, or in both analyses (Fig. S2). This was supported by the Good's coverage parameter (Good, 1953), which never fell below 0.992 and in most cases approximated or reached a value of 1.000 which equals full coverage (Table S3). Interestingly, Namibian soils harboured the largest number of observed OTUs whereas the soils from Costa Rica and Greenland contained the smallest number of observed OTUs among the analysed soils. Using non-parametric richness estimators, a maximum of 83 OTUs at the species level was estimated for Namibian soils whereas the Greenland tundra soil harboured the lowest richness with an estimated maximum of 8 OTUs at the species level (Table S3). Non-parametric richness estimators were previously shown to underestimate OTU numbers because of sensitivity against low coverage as commonly observed in clone libraries (Hong *et al*., 2006). However, the small differences between the observed and estimated OTU richness in all soil samples (on average 5–10 OTUs) indicate that this bias was largely eliminated due to high coverage of all samples in our analysis.

Normalizing samples to an equal sampling depth of 1300 reads per soil and sequencing direction revealed a strong correlation of OTU richness to the total nitrogen and organic carbon content, with highest richness at the lowest total nitrogen and organic carbon content (Fig. S3). A weaker correlation was observed when OTU richness was compared with soil pH (Fig. S3) and no correlation was apparent when OTU richness was compared with the C/N ratio (data not shown).

### Geographically separated soils harbour distinct AOA communities

**Fig. 3:**
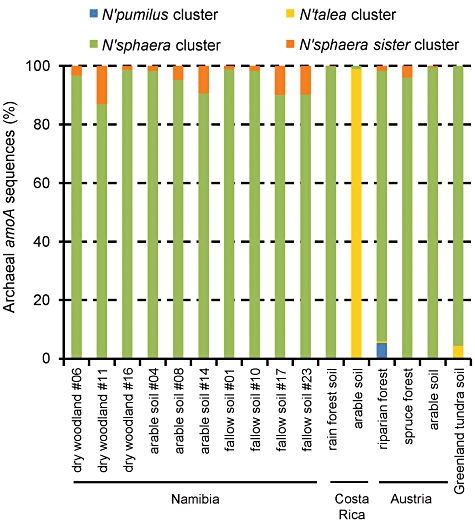
Relative abundance of sequences affiliated with the five major archaeal *amoA* clusters in the analysed soils (no *Nitrosocaldus* cluster representatives were detected). The combined analysis of forward and reverse sequenced archaeal *amoA* gene fragments retrieved by 454 pyrosequencing is shown.

Most (98%) of the soil *amoA* sequences retrieved in this study had a close relative at the species level (≥ 85% sequence identity) in the reference database (Fig. S4) and the largest fraction of these sequences were affiliated to the *Nitrososphaera* cluster (Fig. 3, Table 1). Only 4%, 0.2% and 4% of sequenced *amoA* were distributed among the *Nitrosotalea*, *Nitrosopumilis* and *Nitrososphaera* sister cluster respectively; however, no representatives of the *Nitrosocaldus* cluster were detected in the investigated soils. In this context, it should be noted that the presence of *Nitrosocaldus* cluster representatives in the investigated soils cannot be completely ruled out because of mismatches of the used primers to the *amoA* gene of *Candidatus* Nitrosocaldus yellowstonii (Fig. S5).

**Table 1:** Phylogenetic affiliation of sequenced *amoA* amplicons

			Relative abundance of phylogenetic groups (%)^a^																
			
			Namibia										Costa Rica		Austria			Greenland	
							
Phylogenetic level 1	Phylogenetic level 2	Phylogenetic level 3	Dry woodland #06	Dry woodland #11	Dry woodland #16	Arable soil #04	Arable soil #08	Arable soil #14	Fallow soil #01	Fallow soil #10	Fallow soil #17	Fallow soil #23	Rain forest	Arable soil	Riparian forest	Spruce forest	Arable soil	Tundra	Normalized average^d^
*N'sphaera* sister cluster	No subcluster^b^	No subcluster	–	0	–	0	0	0	–	–	0	0	–	–	–	–	–	–	0
	Subcluster 1	Subcluster 1.1	1	0	1	0	0	1	0	1	1	1	0	–	2	–	0	–	0
	Subcluster 2	No subcluster	2	**13**	1	2	4	8	1	1	9	9	0	–	–	4	0	–	2
	Novel OTUs^c^	Novel OTUs	0	–	0	0	–	0	0	–	–	–	–	–	–	–	–	–	0
*N'sphaera* cluster	No subcluster	No subcluster	0	1	2	1	7	2	2	4	6	3	–	0	**34**	**16**	**61**	–	10
		M'genome fragment 54d9-related	–	0	0	0	1	0	0	0	1	1	0	0	2	1	26	–	2
	Subcluster 1	No subcluster	–	0	–	0	0	–	0	1	0	0	–	–	–	–	0	–	0
		Subcluster 1.1	1	**26**	**94**	7	**35**	**37**	**64**	**49**	**14**	**30**	0	0	1	–	**29**	0	11
	Subcluster 2	subcluster 2.1	0	0	0	–	0	0	1	1	0	0	–	–	0	–	1	–	0
	Subcluster 3	Subcluster 3.1	**23**	**47**	1	**75**	**30**	**33**	19	**26**	**58**	**45**	0	–	–	–	–	–	9
		Subcluster 3.2	**61**	3	–	7	3	1	0	0	4	3	0	0	–	0	–	–	2
		Subcluster 3.3	1	0	0	1	0	1	0	0	2	1	–	–	–	–	–	0	0
	Subcluster 4	No subcluster	–	0	–	0	0	0	0	0	–	0	–	–	0	3	–	–	0
		Subcluster 4.1	–	0	0	0	–	–	0	0	–	0	0	–	**46**	1	1	–	4
	Subcluster 5	No subcluster	–	–	–	–	–	–	–	–	–	–	–	0	–	–	–	–	0
		Subcluster 5.1	1	–	–	–	0	–	–	–	–	–	0	–	–	**63**	–	–	5
	Subcluster 6	Subcluster 6.1	–	1	1	–	1	1	1	1	0	2	–	–	2	–	4	–	1
	Subcluster 7	No subcluster	0	–	–	–	–	–	–	–	–	–	–	–	0	–	–	**95**	24
		Subcluster 7.1	4	–	–	–	0	–	–	–	–	–	–	–	–	–	–	–	0
		Subcluster 7.2	2	–	–	–	0	–	0	–	–	0	**99**	0	–	0	–	–	12
	Subcluster 8	Subcluster 8.1	1	0	0	0	0	0	–	0	0	–	–	0	0	–	3	–	0
		Subcluster 8.2	–	–	–	–	–	–	–	–	–	–	–	–	0	1	–	0	0
	Subcluster 9	No subcluster	0	6	1	2	**17**	**10**	**10**	**15**	4	4	–	–	8	6	0	–	3
	Subcluster 11	No subcluster	0	0	–	0	–	0	–	0	0	0	–	–	–	–	–	0	0
	Novel OTUs^c^	Novel OTUs	3	2	0	5	1	5	2	1	1	2	0	0	1	7	0	–	1
*N'talea* cluster	Subcluster 1	Subcluster 1.1	–	–	–	–	–	–	–	–	–	0	0	**99**	–	0	–	5	14
	Subcluster 2	No subcluster	–	–	–	–	–	–	–	–	–	–	–	–	1	–	–	–	0
*N'pumilus* cluster	Subcluster 1	Subcluster 1.1	–	–	–	–	–	–	–	–	–	–	–	–	1	–	–	–	0
	Subcluster 5	Subcluster 5.1	–	–	–	–	–	–	–	–	–	–	–	–	0	–	–	–	0
		Subcluster 5.2	–	–	–	–	–	–	–	–	–	–	0	0	–	–	–	–	0
	Subcluster 15	No subcluster	–	–	–	–	–	–	–	–	–	–	–	0	4	–	0	–	0

Subclusters with ≥ 10% relative abundance are given in bold.

a. ‘0’ represents phylogenetic groups which had a relative abundance between true 0% and 0.5%; ‘–’ represents phylogenetic groups which had a relative abundance of true 0%.

b. *amoA* amplicons that shared ≥ 85% sequences identity (species-level) to a database *amoA* sequence, which did not fall into a stable subcluster, kept the cluster affiliation of the higher phylogenetic level.

c. 454 *amoA* amplicons with < 85% sequence similarity to known archaeal *amoA* and falling into one of the archaeal *amoA* clusters as revealed by phylogenetic tree reconstructions.

d. The average detection of the various subclusters was normalized against the different sample numbers of the various geographic locations.

About 2% of the sequences (*n* = 1832) represented novel *amoA* at the species level, but were also all affiliated with the *Nitrososphaera* or *Nitrososphaera* sister cluster as determined by phylogenetic analysis (exemplified in Fig. S6B). In total, 13 and 30 novel OTUs at the species level were detected in the forward and reverse analysis respectively (Table S6). Representing sequences of forward and reverse OTUs that overlapped by more than 260 nt (arbitrarily chosen) and shared at least 97% sequence similarity (within the 454 sequencing error range, Kunin *et al*., 2009) were merged to represent one OTU. This resulted in six merged OTUs that reduced the number of potentially novel AOA in the pyrosequencing data to 30–37 OTUs at the species level (the range results from forward and reverse OTUs that could not be merged but potentially might represent the same OTU). Interestingly, merged OTU2 and reverse OTU03 reached relative abundances of up to 5% in Namibian and Austrian soils respectively. However, most of the novel *amoA* OTUs were of minor abundance (Fig. S6A).

The individual soils were dominated by *Nitrososphaera* cluster *amoA*, with the only exception being an arable soil from Costa Rica that was dominated by *Nitrosotalea* cluster *amoA* (Fig. 3, Table 1). An overrepresentation of the *Nitrosotalea* cluster in this soil due to 454 sequencing biases (Gomez-Alvarez *et al*., 2009) is unlikely as this result was obtained independently in the forward and reverse sequencing analysis. Although *Nitrososphaera* sister cluster *amoA* never exceeded 13% of all *amoA* sequences per soil, they could be detected in most of the analysed soils with the exception of the Greenland tundra soil and the arable soil from Costa Rica. In contrast, *Nitrosopumilus* cluster *amoA* were absent or extremely rare in all soils, with the only exception being an Austrian riparian soil where 5% of the sequences could be assigned to this cluster (Fig. 3, Table 1).

**Fig. 4:**
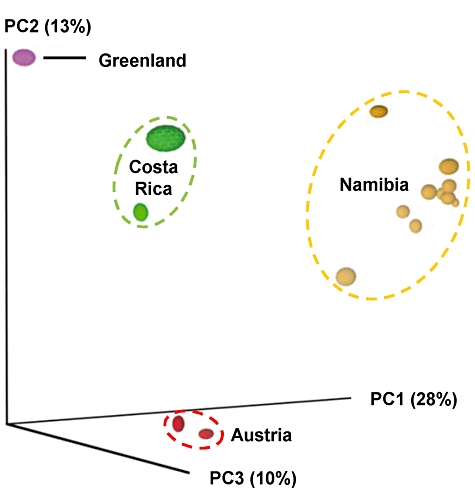
Principal component analysis based on presence/absence of OTUs (jackknifed unweighted UniFrac) and separating soils according to their geographic origin. For this analysis, observed *amoA* OTUs at 97% sequence identity were used (representing the highest possible phylogenetic resolution) and normalized to 1300 reads per soil and sequencing direction. The Austrian spruce forest soil was omitted from the analysis due to a sequence number of less than 1300 reads. Analysis of the forward sequences is shown; analysis of reverse sequences gave similar results (data not shown).

There were considerable differences in AOA community composition between individual soils within the *Nitrososphaera* cluster (Table 1). Based on mere presence/absence of OTUs at 97% sequence identity (unweighted UniFrac), individual soils were best separated according to their geographic origin i.e. the four countries on different continents (Fig. 4). This strong correlation was supported by partial Mantel regression, which determines the spatial variability in species composition after removing the effects of environmental variables (R = 0.64–0.72, *P* = 0.001, Table S4). Here, soils from the same geographic origin were treated as replicates. Statistical analysis for the association strength of specific OTUs to geographic location was tested by determination of the indicator value for each OTU-soil origin combination (De Caceres and Legendre, 2009). OTUs that were exclusively detected in one of the individual geographic locations (indicator value of 1.000) were mainly affiliated to *Nitrososphaera* subcluster 1.1 and 3.1 for Namibian soils, *Nitrososphaera* subcluster 7.2 and *Nitrosotaleus* subcluster 1.1 for Costa Rican soils, and *Nitrososphaera* cluster 4.1 and OTUs not resolved in any subcluster within the *Nitrososphaera* cluster, which included OTUs closely related to soil metagenome fragment 54d9, for Austrian soils (Table S5). No statistical testing could be performed for Greenland soils, because only one tundra soil was investigated. However, this soil was dominated by *Nitrososphaera* subcluster 7 representatives (Table 1).

Considering OTU abundance in weighted UniFrac analysis revealed that individual soils were separated best according to their combined total nitrogen/organic carbon content or to their pH (Fig. S7). The C/N ratio had no effect on community composition (data not shown). Mantel regression analysis, which treated soils with similar soil parameters as replicates, supported these results and revealed that the combination of all determined soil parameters gave the strongest correlation (R = 0.59–0.60, *P* = 0.01), whereas correlations to single soil parameters were less pronounced (Table S4). This explains why individual soils could be simultaneously separated according to different soil parameters in the weighted UniFrac analysis and showed that their combined influence was the best predictor for OTU abundance in individual soils. For Namibian soils sharing a similar soil pH, an increase in indicator OTUs affiliated to *Nitrososphaera* subcluster 1.1 and 9 was observed (Table S5), which partially explains why the acidic Namibian dry woodland soil #06 was separated from other Namibian soils in UniFrac analyses. Also three indicator OTUs within the *Nitrososphaera* subcluster 1.1 and 8.1 were specific for an intermediate total nitrogen and organic carbon content but showed no affiliation to any geographic region. The remaining indicator OTUs for soil parameters overlapped with indicator OTUs for geographic location (Tables S5 and S7).

Additional mantel regression testing for correlations between the AOA community and the type of land management in the Namibian soil subset revealed no significant differences (data not shown).

## Discussion

In this study, we assembled an encompassing high-quality archaeal *amoA* database composed of systematically selected reference sequences representing the recognized archaeal *amoA* diversity at a 97% sequence identity level (available for download: File S1). Furthermore, a consensus phylogeny based on this widely used functional marker gene was established by applying three different phylogeny inference methods and a nomenclature system for all well-supported clusters was developed. Known archaeal *amoA* sequences diversify into five major clusters: the previously observed (albeit named differently) *Nitrososphaera*, *Nitrosopumilus*, *Nitrosotalea* and *Nitrosocaldus* clusters as well as a newly recognized sister group of the *Nitrososphaera* cluster (Fig. 1A). This sister group of the *Nitrososphaera* cluster is not yet represented by a cultured AOA or a metagenome fragment and consistent with our nomenclature of the other clusters, we propose to re-name it as soon as such a culture will be published. We introduced a new nomenclature system for the AOA lineages as (i) different names were used in the past for identical lineages (e.g. soil lineage and cluster I.1b), (ii) the previously called soil and marine lineages contain many sequences from other environments and thus these names are misleading, and (iii) the frequently used numbering system (I.1a, I1b, etc.) is more difficult to memorize and integration of newly recognized lineages that are sister groups to recognized lineages would result in long abbreviations (e.g. the *Nitrosopumilus* cluster and the recently discovered *Nitrosotalea* cluster would have to be re-named in lineage I.1a-1 and I.1a-2). The latter point is also important because it seems very likely that additional major lineages of AOA exist as previous 16S rRNA-based studies have identified additional clades within the *Thaumarchaeota* whose abundances were correlated to archaeal *amoA* copy numbers (which were not sequenced) or ammonium concentrations (Kemnitz *et al*., 2007; Mincer *et al*., 2007).

Analogous to the approach applied by Purkhold and colleagues (2000) for ammonia-oxidizing bacteria, we determined for AOA an *amoA* species-level sequence identity threshold (Fig. 2). Application of the inferred 85% identity threshold demonstrated that the entire *amoA* data set contained at least 83 different AOA species, which is an order of magnitude higher than the eight described Candidatus species from this guild and illustrates that in contrast to AOB the vast majority of AOA species have not yet been successfully cultured. Interestingly, our results from the PCR-based 454 pyrosequencing AOA diversity survey suggest that the assembled archaeal *amoA* database (although not including sequences from environmental diversity surveys deposited after June 2010) is already rather representative of the species-level diversity of this guild in terrestrial systems. Only 2% (*n* = 1832) of the obtained *amoA* sequences from the 16 analysed soils represented new species-level OTUs (Fig. S4) increasing the number of such units to 113–120. In this context, it is particularly noteworthy that none of the newly detected AOA species-level OTUs was found in an abundance above 5% in any of the analysed soils (Fig. S6A). This demonstrates that close relatives of all numerically dominant AOA in these samples were already represented in the database. Similar results were obtained for methanotrophic bacteria, where deep sequencing of *pmoA* genes (coding for the α-subunit of the particulate methane monooxygenase) revealed that the existing *pmoA* database covers most of the *pmoA* diversity retrieved by pyrosequencing from *Sphagnum* mosses and rice paddy soils (Kip *et al*., 2011; Lüke and Frenzel, 2011). However, the limited phylogenetic novelty of the detected AOA in the 16 soils could theoretically also have been caused by incomplete coverage of the actual archaeal *amoA* diversity by the applied PCR primers. While perfect coverage of the natural diversity of a gene by any PCR primer pair can never be guaranteed, a recent specificity check of our primers revealed that – in contrast to the frequently applied primers Arch-amoF/Arch-amoR (Francis *et al*., 2005) – they fully match all *amoA* genes for which sequence information in the target region is available (Fig. S5). The only exception is the *amoA* gene of *Nitrosocaldus yellowstonii*, which possesses mismatches to the used primers and thus might have led to the lack of *amoA* sequences from the *Nitrosocaldus* cluster in our samples.

Fifteen of the 16 analysed soils were clearly dominated by members of the *Nitrososphaera* cluster (Fig. 3), which is in good agreement with (i) previous soil archaeal *amoA* diversity studies (e.g. Francis *et al*., 2005; Leininger *et al*., 2006; Nicol *et al*., 2008; Wessen *et al*., 2011), (ii) the documented metabolic activity of representatives of this cluster in soils (Pratscher *et al*., 2011), and (iii) with results from a global survey of archaeal diversity using high-throughput sequencing of 16S rRNA genes (Bates *et al*., 2011). In the latter study, group I.1b archaea, a synonym for the *Nitrososphaera* cluster, were dominating in a selection of 146 soils covering different biomes like forest, grasslands, deserts and agricultural soil. Consistent with our data, only few soils of this extensive 16S rRNA gene-based study harboured minor populations of group I.1a archaea, representing the *Nitrospumilus* cluster (Bates *et al*., 2011). This also coincides with the low abundance or absence of the *Nitrosopumilus amoA* cluster in soils reported by other studies (e.g. Nicol *et al*., 2008; Wessen *et al*., 2011).

Interestingly, in our study one acidic arable soil from Costa Rica (pH 4.99) was not dominated by the *Nitrososphaera* cluster but contained almost exclusively members of the *Nitrosotalea* cluster (Fig. 3), which encompasses the first cultured obligate acidophilic AOA (Lehtovirta-Morley *et al*., 2011). However, four other soils in our study with similar pH (4.4–5.1, Table S2) were not characterized by high numbers of the *Nitrosotalea* cluster (Fig. 3) but were rather dominated by subclusters of the *Nitrososphaera* cluster (Table 1), which thus likely harbours additional acidophilic AOA.

The archaeal 16S rRNA gene-based diversity survey mentioned above (Bates *et al*., 2011) showed that only two phylotypes (at 97% sequence identity) belonging to the *Nitrososphaera* cluster constituted > 70% of the retrieved archaeal sequences in 146 analysed soils. The most abundant phylotype showed 97% sequence identity to the 16S rRNA sequence of soil metagenome fragment 54d9 (Treusch *et al*., 2005) and was found in 50% of the analysed soils representing 46% of all retrieved archaeal sequences (range 0–83%, Bates *et al*., 2011). In comparison, in our study sequences that had the *amoA* gene of soil metagenome fragment 54d9 as most similar sequence (93–97% sequence identity) were widely distributed as well (detected in 75% of the analysed soils) but their average abundance was much lower (2%, range 0–26% per soil, Table 1). This possibly reflects that the assignment of environmentally retrieved sequences was performed with higher phylogenetic resolution in our study. Interestingly, in soils analysed in this study members of the *Nitrososphaera* subcluster 1.1, which includes the cultured representatives *Candidatus* Nitrososphaera gargensis (Hatzenpichler *et al*., 2008) and *Candidatus* Nitrososphaera viennensis (Tourna *et al*., 2011), were widespread (found in 94% of analysed soils) and often abundant (average 11%, range 0–94%, Table 1).

Analysis of factors that shape AOA community structure in the analysed soils revealed a strong effect of geographic location on the continental scale, which very likely includes also the different climatic conditions of the distant locations (Fig. 4). Despite the fact that co-correlation of geographic locations to undetermined soil parameters can never be completely ruled out, our results indicate that geographic relatedness has a strong effect on the mere presence or absence of different AOA taxa in individual soils. Similar results were previously obtained for other groups of microorganisms, showing that on the scale of thousands of kilometres, historical separation due to mutation, genetic drift or differential selective pressures in the past can counteract forces of dispersal and homogenizing effects of environmental factors (reviewed in Martiny *et al*., 2006). Building on these geographically determined AOA seed banks (Pedrós-Alió, 2006), total nitrogen concentration, organic carbon content, and pH were identified in our study as potential drivers of AOA community composition in the analysed soils (Fig. S7). Besides influencing abundance of individual AOA taxa, theses soil parameters apparently also had an impact on overall AOA species richness in the analysed soils (Fig. S3), although we cannot exclude if an unknown co-correlating factor in the Namibian soils biased these results.

In conclusion, this study provides (i) a systematically assembled archaeal *amoA* reference database covering the recognized diversity of members of this guild at the 97% sequence identity level, (ii) a robust *amoA*-based consensus phylogeny for AOA, which resulted in the description of a new major cluster, and (iii) a new nomenclature system for the evolutionary lineages within the AOA *amoA* tree as resources for future evolutionary and ecological studies of AOA. We show that the current collection of archaeal *amoA* sequences including the newly determined sequences in this study represents at least 113–120 AOA species-level OTUs. Deep sequencing of the archaeal *amoA* genes of 16 different soils revealed that AOA richness can be adequately covered with an easily achievable sequencing effort and ranges from 8–83 species-level OTUs per soil. Furthermore, the pyrosequencing data revealed that our current perception of terrestrial AOA diversity is already surprisingly complete at the species level and that thus the time is ripe for exploring factors driving AOA species richness and community composition (a topic which we only partially addressed in our study due to the relatively limited number of analysed soils). With the exception of a single acidic soil, all analysed soils were dominated by representatives of the *Nitrososphaera* cluster. However, the majority of these soil *amoA* sequences were affiliated to subclusters without any cultured representative, clearly demonstrating the need for future cultivation efforts end ecophysiological studies in order to better understand the ecology of these important nitrifiers.

## Experimental procedures

### Generating an encompassing *amoA* reference database

A reference database containing all publicly available archaeal *amoA* sequences was built by tblastx analysis (Camacho *et al*., 2009). To define a bit score threshold for retrieving archaeal *amoA* from public databases, each entry of an archaeal *amoA* in-house ‘seed’ database (*n* = 1516) was blasted against all other in-house archaeal *amoA*, bacterial *amoA* and *pmoA* (including type I and II methanotrophs as well as *Crenothrix polyspora*, *Methylacidiphilum kamchatkense* and *Methylacidiphilum infernorum*) sequences, with bacterial monooxygenase genes serving as outgroup. The highest bit score of the outgroup entries (*n* = 1819) + 10% (to make the search more conservative) was then used as a bit score threshold for the blast search. This threshold was determined for each in-house archaeal *amoA* entry separately. Thereafter, each archaeal *amoA* entry with its own threshold was blasted one by one against NCBI's non-redundant and environmental databases (http://www.ncbi.nlm.nih.gov), the IMG/M database (http://www.jgi.doe.gov) and the Camera database (http://camera.calit2.net) (status June 2010).

Newly retrieved archaeal *amoA* sequences were compared with the in-house ‘seed’ database using CD-HIT-EST-2D (Huang *et al*., 2010). All sequences which showed ≥ 97% nucleic acid sequence identity with a database entry or were shorter than the shortest sequence in the ‘seed’ database were not considered further in order to reduce sequence redundancy and to keep only sequences suitable for phylogenetic analysis. In a second step, remaining sequences and sequences of the in-house database were clustered in parallel with standard CD-HIT-EST (Huang *et al*., 2010) on a 98.5% identity threshold over ≥ 97% of the smaller sequence resulting in clusters of ≥ 97% sequence identity (for details please refer to Huang *et al*., 2010). Representing sequences of the generated clusters covered the complete archaeal *amoA* diversity by June 2010. Thereafter, cluster representatives of newly retrieved *amoA* were aligned one after the other to representatives of the in-house database using Muscle (using the -profile option and a gap open score of −750). To avoid frame shifts in these publically available sequences due to sequencing errors, bases which introduced gaps in the existing alignment that were not a multiple of three were removed from the respective sequences after alignment using an in-house script (116 bases in 21 sequences). This procedure discriminated against pseudogenes with true frame shifts, which however should be much rarer than deposited sequences with sequencing errors. As a further quality control step, alignments were inspected manually and representing sequences shorter than 592 nt or with internal stop codons were removed. All cluster representatives of newly retrieved sequences represented archaeal *amoA* with no falsely recovered bacterial *amoA* genes or *pmoA* genes of methanotrophic bacteria as revealed by phylogenetic analysis (detailed below).

### Reconstruction of archaeal *amoA* phylogeny

Phylogenetic inference analysis of archaeal *amoA* sequences in the constructed reference database was done using 592 unambiguously aligned nucleotides. For phylogeny reconstruction, nucleic acid sequences were preferred over deduced amino acid sequences because of their higher phylogenetic resolution. Phylogenetic trees were reconstructed using (i) the neighbour joining algorithm based on a Jukes-Cantor corrected distance matrix within the Phylip package (Felsenstein, 1989), (ii) the maximum parsimony algorithm based on a transversion–transition matrix that assigns two times the cost for a transition compared with a transversion within the PAUP package v 4.0 (Swofford, 2003), and (iii) the maximum likelihood algorithm of the RAxML v7.2.8 package (Stamatakis, 2006). A consensus tree using the majority rule was constructed from the different treeing methods (Phylip). Branch lengths of the consensus tree were inferred by the Fitch algorithm using a Jukes-Cantor corrected distance matrix (Phylip). Local changes of multifurcations to bi-furcations in the consensus tree, which were in some cases introduced by the Fitch algorithm, were manually corrected. Subsequently, three phylogenetic levels were defined by following the first, second and third (multi)-furcation starting from the root. A running number was assigned to each cluster that consisted of more than three sequences at the second and third phylogenetic level (Fig. 1, Fig. S1). *amoA* sequences from AOA cultures or enrichments, which were published after this time-consuming analysis (Blainey *et al*., 2011; Kim *et al*., 2011; Lehtovirta-Morley *et al*., 2011), were added to the trees using the parsimony interactive tool of ARB (Ludwig *et al*., 2004). An ARB database containing all *amoA* representatives and the consensus tree can be found in File S1.

### Analysed soils and molecular analysis

Analysed soils are listed together with geographic coordinates and determined soil parameters in Table S1. Soil sampling, soil parameter determination, DNA extraction, PCR amplification, ligation of barcodes to PCR amplicons and 454 pyrosequencing followed standard protocols and are described in detail in Appendix S1 (*Supporting Methods*).

### Bioinformatic analysis

If not stated otherwise, bioinformatic analysis was performed using the Mothur software package (http://www.mothur.org, Schloss *et al*., 2009). Raw 454 sequences were quality screened and trimmed using Lucy 1.20 (Chou and Holmes, 2001) keeping sequences of ≥ 250 nt which had an average Phred score of ≥ 27; if required low quality parts of the sequences were trimmed until the remaining sequences obeyed these criteria. Thereafter, 454 sequences were screened for their barcode and primer sequences keeping only sequences with exact matches.

454 sequencing errors were further minimized in a stepwise procedure. Initially, high-quality sequences identified by the above described selection procedure were pre-clustered using the pre.cluster-function in Mothur, which is less computationally intensive than CD-HIT clustering and identifies potential sequencing errors. Pre.cluster ranks identical sequences in order of their abundance and assigns less abundant sequences to more abundant sequences using a maximum of n mismatches by assuming that the probability of a 454 sequencing error to occur is higher in less abundant sequences. In this study, n equaled 3 which corresponds to a sequence identity of ≥ 97.6% at a sequence length ≥ 250 nt. Representatives of the pre.cluster step were further grouped using CD-HIT-EST clustering (Huang *et al*., 2010) at a 98.5% sequence identity level over 97% of the smaller sequence, which results in clusters of ≥ 97% sequence identity with the longest sequence as representative of each cluster (for details please refer to Huang *et al*., 2010). Thereafter, representing sequences of all CD-HIT clusters were screened for frame shifts (most likely caused by 454 sequencing errors) using an in-house adaptation of FrameD (Schiex *et al*., 2003). Because FrameD identifies the region of the frame shift but not the exact deleted or inserted base, deletions and neighbouring bases were masked by ‘N's and insertions were removed and the in-frame base was masked by an N.

To screen for chimeras two procedures were applied. Initially, all CD-HIT cluster representatives were aligned one-by-one with Muscle (using the -profile option and a gap open score of −750) to the *amoA* reference database and their sequence dissimilarity to the next relative was determined. The alignments of those sequences with highest dissimilarities (21–35%) were manually inspected, which resulted in six detected chimeras that did not span the entire amplicon length but instead had sequence information upstream of the forward primer or downstream of the reverse primer. After initial chimera removal, representatives were aligned against the reference database using k-mer searching of 8mers and manually screened for alignment errors. During this process, 21 representing sequences were identified as chimeras due to massive misalignment of certain sequence regions. This was verified by blasting putative chimeras (blastn) against NCBI's non-redundant database (http://www.ncbi.nlm.nih.gov) and retrieving a clear break in the local alignment, which affiliated the queried chimera to two very distinct sequence entries in NCBI's database. Identified chimeras were either discarded completely from further processing or manually trimmed to remove the chimeric sequence part if the remaining sequence was ≥ 250 nt. Thereafter, sequences were grouped based on their sequencing direction (forward or reverse) and subjected to rarefaction, binning into OTUs, and α-diversity analysis.

Phylogenetic assignment of high-quality 454 sequences was performed by aligning cluster representatives (97% sequence identity) individually to the *amoA* reference database using Muscle (Edgar, 2004) and determining the most similar reference database entry down to an *amoA* sequence identity of 85% (Table S8). 454 sequences with < 85% identity to a reference database entry were binned separately and clustered at 85% sequence identity. The phylogenetic position of representing sequences of novel *amoA* OTUs within the *amoA* consensus tree was deduced by two independent inference methods: (i) the interactive parsimony tool within the ARB software package (Ludwig *et al*., 2004) and (ii) and a distance matrix method (neighbour joining tree based on a Jukes-Cantor corrected distance matrix).

### Statistical analysis

Statistical analysis was performed separately on forward and reverse sequenced OTUs at the 97% identity level. Jackknifed weighted and unweighted UniFrac distance metrics (1300 forward or 1300 reverse sequenced reads per soil) and corresponding principal component analysis plots were generated within the Qiime software package (Caporaso *et al*., 2010). Further statistical analyses were performed in the R statistical software package (R-Development-Core-Team, 2010) with soils of the same geographic location or with similar chemical parameters treated as replicates (defined as soil groups). The ecodist package (Goslee and Urban, 2007) was used for Mantel's test. For partial Mantel regression, geographic distances were log-transformed [ln(1 + *x*)]. The indicspecies package (De Caceres and Legendre, 2009) was used for indicator OTU analysis. An indicator value (range: 0–1) was generated for each OTU-soil group combination using presence/absence of OTUs; 999 bootstraps were applied to generate a *P*-value for each indicator value. Uncorrected *P*-values are given in the indicator OTU analysis since testing of approximately 1000 OTUs results in excessively overcorrected *P*-values, which would lead to the rejection of all indicator OTUs. To avoid reporting false positives, only indicator OTUs with an indicator value of 1.000 were reported, which means that these OTUs were only detected in the group of soils for which they serve as indicators.

### Deposited 454 read accession numbers

Sequences were submitted to the Sequence Read Archive (SRA) at NCBI under the accession number SRA047303.

## Supporting information

**Fig. S1.** Consensus tree illustrating thediversification of the *Nitrosopumilus* cluster at the secondand third phylogenetic level. The tree was determined using 592unambiguously aligned positions of 735 nucleic acid sequences thatevenly cover the known sequence space of archaeal *amoA* at a≥ 97% sequence identity level. Numbers in circles represent thesecond phylogenetic level (e.g. *Nitrosopumilus* subcluster1), whereas the third phylogenetic level is directly indicated atthe tree branch (e.g. *Nitrosopumilus* subcluster 1.1); sequences that did not form stable sublineages of more than three representatives kept the affiliation of the higher phylogenetic level and are indicated by their NCBI accession number. The consensus tree and the source alignment of representing sequences can be found in File S1. The scale bar indicates 10% estimated sequence divergence.

**Fig. S2.** Rarefaction analysis of forward andreverse sequenced *amoA* at the species level cut-off of 85%*amoA* identity.

**Fig. S3.** Correlation analysis of totalnitrogen, organic carbon and soil pH to OTU richness at the specieslevel cut-off of 85% *amoA* identity when normalized to 1300reads per soil and sequencing direction. The Austrian spruce forestwas omitted from the analysis due to a sequence number of less than1300 reads. The analysis of the reverse sequences is shown with allcorrelations having a *P*-value < 0.05; analyses of forwardsequences gave similar results (data not shown). Highest AOAspecies richness was observed at the lowest total nitrogen andorganic carbon content, which agrees well with the cumulativerecent findings that AOA are generally adapted to low ammoniaconcentrations and are inhibited by high loads of dissolved organiccarbon (Könneke *et al*., 2005; Hatzenpichler *etal*., 2008; de la Torre *et al*., 2008; Erguder *etal*., 2009; Martens-Habbena *et al*., 2009; Di *etal*., 2010; Pratscher *et al*., 2011; Verhamme *etal*., 2011). The few detected AOA species in soils with highloads of nitrogen and organic carbon indicate the existence ofecotypes adapted also to these conditions or represent AOA thatperform a mixotrophic or heterotrophic lifestyle (Hallam *etal*., 2006; Jia and Conrad, 2009; Mußmann *et al*.,2011; Tourna *et al*., 2011). For soil pH, AOA speciesrichness followed the general trend of microbial species richnessobserved in a large survey of soils (Lauber *et al*., 2009),with a maximum of species at slightly acidic pH (pH = 6).

**Fig. S4.** Abundance plot showing sequenceidentities of soil archaeal *amoA* retrieved by 454pyrosequencing to next relatives in public databases. Theapproximate species-level threshold of 85% *amoA* sequence identity is indicated by a dotted line.

**Fig. S5.** *In silico* specificity analysisof the archaeal *amoA* primers used in this study against allarchaeal *amoA* sequences covering the primer target regions.The primer regions of *Candidatus* Nitrosotalea devanaterra,*Candidatus* Nitrosocaldus yellowstonii and *Candidatus*Nitrosotenuis uzonensis (affiliated to *Nitrosopumilus*subcluster 5.1) represent partly unpublished data and were kindlyprovided by Graeme Nicol (Institute of Biological and EnvironmentalSciences, University of Aberdeen), José de la Torre (Department of Biology, San Francisco State University) and Roland Hatzenpichler and Susanne Haider (Department. of Microbial Ecology, University of Vienna) respectively.

**Fig. S6.** Analysis of novel *amoA* thatshared less than 85% sequence identity to deposited sequences inpublic databases.

A. Abundance of novel OTUs at the species levelcut-off of 85% *amoA* identity in the respective samples.Representing sequences of forward and reverse OTUs that overlappedby more than 260 nt and shared at least 97% sequence similarity(within the 454 sequencing error range, Kunin *et al*., 2009)were merged to represent one OTU. A detailed list of novel OTUsincluding their representing sequence is given in Table S6.

B.Phylogenetic position of selected novel *amoA* usingrepresenting sequences of all merged OTUs. The phylogeneticposition of representing sequences of novel *amoA* OTUs withinthe *amoA* consensus tree was deduced by two independentinference methods: (i) the interactive parsimony tool within theARB software package (Ludwig *et al*., 2004) and (ii) and adistance matrix method (neighbour joining tree based on aJukes-Cantor corrected distance matrix). Thereafter, novel*amoA* OTU representatives were added manually to the archaeal*amoA* consensus tree (Fig. 1) without changing the overall tree topology (as indicated by the dotted branches of uniform length of the added sequences).

**Fig. S7.** Principal component analysis based onOTU abundance (jackknifed weighted UniFrac) and separating soilsaccording to their combined total nitrogen/organic carbon contentor to soil pH. For this analysis, observed *amoA* OTUs at 97% sequence identity were used (representing the highest possible phylogenetic resolution) and normalized to 1300 reads per soil and sequencing direction. The Austrian spruce forest soil was omitted from the analysis due to a sequence number of less than 1300 reads. Analysis of the forward sequences is shown; analysis of reverse sequences gave similar results (data not shown).

**Table S1.** Highest nucleic acid sequenceidentity between representing sequences of the major archaeal*amoA* clusters. Presented identities were not corrected by substitution models.

**Table S2.** Soil samples and determined soil parameters.

**Table S3.** Sequencing results and number ofobserved and estimated OTUs at the species level (85% *amoA* identity).

**Table S4.** Correlation between beta diversity ofsites (unweighted and weighted UniFrac) and measured soilcharacteristics as determined by Mantel&apos;s test. Determinedparameters are averages based on 100 jackknifed OTU tablesnormalized to 1300 *amoA* sequences per soil sample.

**Table S5.** Number of indicator OTUs (97%*amoA* identity) in different *amoA* lineages. Only OTUs with an indicator value of 1.000 (exclusively detected under a certain tested soil characteristic) were summarized. A detailed list of individual indicator OTUs with their next relatives in public databases is given in Table S7. dw: dry weight. N, total nitrogen (% dry weight); C, total org. carbon (% dry weight).

**Table S6.** Representing sequences and totalretrieved sequences reads of novel archaeal *amoA* OTUs at the species level cut-off of 85% identity in the individual analysed soil samples.

**Table S7.** Indicator OTU analysis for geographic location, pH, and combined total N and organic carbon content of soils.

**Table S8.** OTU classification of *amoA* sequences at 97% identity. For each OTU the representing sequence, its affiliation, and the name and distance to the next relative in public databases is given.

**File S1.** Archaeal *amoA* ARB databaseencompassing the consensus tree and the source alignment ofsequences representing clusters at ≥ 97% sequence identity ofall publicly available archaeal *amoA* genes by June 2010.
