# Supplementary material for: amoA-based consensus phylogeny of ammonia-oxidizing archaea and deep sequencing of amoA genes from soils of four different geographic regions
Source: Environ Microbiol. 2012 Feb;14(2):525–39. doi: 10.1111/j.1462-2920.2011.02666.x (PMC3328746; doi:10.1111/j.1462-2920.2011.02666.x)
Supplement: Supplementary file 2 [file emi0014-0525-SD2.pdf]

Supporting Information for

***amoA*-based consensus phylogeny of ammonia-oxidizing archaea and deep sequencing of *amoA* genes from soils of four different geographic regions**

Michael Pester[1], Thomas Rattei[2], Stefan Flechl[1], Alexander Gröngröft[3], Andreas Richter[4], Jörg Overmann[5], Barbara Reinhold-Hurek[6], Alexander Loy[1], and Michael Wagner[1*]

[1]*Department for Microbial Ecology, University of Vienna, Althanstrasse 14, A-1090 Vienna, Austria*

[2]*Department for Computational Systems Biology, University of Vienna, Althanstrasse 14, A-1090 Vienna, Austria*

[3]*Institute of Soil Science, University of Hamburg, Allende-Platz 2, D-20146 Hamburg, Germany*

[4]*Department of Chemical Ecology and Ecosystem Research, University of Vienna, Althanstrasse 14, A-1090 Vienna, Austria*

[5]*DSMZ, Inhoffenstraße 7B, D-38124 Braunschweig, Germany*

[6]*Department of Microbe-Plant Interactions, University of Bremen, Postfach 330440, D-28334 Bremen, Germany*

*Corresponding author: Department for Microbial Ecology, University of Vienna, Althanstrasse 14, A-1090 Vienna, Austria, Phone: +43 1 4277 54390, Fax: +43 1 4277 54389, wagner@microbial-ecology.net

Supporting Methods

Soil sampling

All soil samples were taken from the surface layer (0–15 cm) after removing litter and large roots. Namibian soils were sampled in March 2007 and preserved in RNALater (Applied Biosystems/Ambion, Austin, TX, USA) until DNA extraction with storage at ambient temperature in the field and at –20 °C in the laboratory. Soils from Greenland, Austria, and Costa Rica were sampled in August, October, and November 2008, respectively, and stored at ambient temperature in the field and at –20 °C in the laboratory until DNA extraction. Total organic carbon and nitrogen in soils were determined by elemental analysis (EA 1110, CE Instruments, Hindley Green, UK) and pH was measured in a 10 mM $CaCl_2$ soil suspension.

DNA extraction, PCR, barcoding of amplicons, and 454 pyrosequencing

DNA was extracted in duplicates from Namibian soils and in triplicates from all remaining soils using 0.25 g of soil and the PowerSoil DNA Extraction Kit (Mo Bio Laboratories, Inc., Carlsbad, CA, USA). Namibian soils were washed twice with phosphate-buffered saline solution (0.9 mM NaH_2PO_4, 3.6 mM Na_2HPO_4, 130 mM NaCl, pH 7.2–7.4) before DNA extraction to remove RNALater from samples as recommended by the manufacturer. Each replicate DNA extract was used to amplify an approximately 629-bp long fragment of the archaeal *amoA* using the primers CamoA-19f (5'- ATG GTC TGG YTW AGA CG -3') and CamoA-616r (5'- GCC ATC CAB CKR TAN GTC CA -3') (both modified by Maria Tourna from Tourna *et al.,* 2008). An *in silico* check for primer specificity revealed that the used primers match all currently available archaeal *amoA* sequences covering the primer region except for the *amoA* gene of *Candidatus* Nitrosocaldus yellowstonii (Fig. S5). The following PCR reaction mixture was applied: 1 µM of each primer, 1.5 mM $MgCl_2$, 0.2 mM of each dNTP, 10 ng μl^{-1} bovine serum albumin, 5 U Taq DNA Polymerase and 1× reaction buffer [all reagents were from Fermentas, St. Leon-Rot, Germany, except primers (Thermo

Scientific, Ulm, Germany)]. Template DNA was initially denatured at 95°C for 5 min followed by 30 cycles of denaturing (95°C, 30 s), primer annealing (50°C, 1 min), and elongation (72°C, 1 min) and a final 5 min extension at 72°C. To capture as much diversity as possible, the lowest annealing temperature giving a single PCR product of the expected size was determined in a temperature gradient PCR using a selected soil DNA extract as template. Replicate PCR products were checked for the correct length by standard agarose gel electrophoresis, pooled per soil sample, and purified using the MinElute PCR Purification Kit (QIAGen, Hilden, Germany). To distinguish *amoA* amplicons originating from different soil samples, barcode oligonucleotides of 7 bp in length were ligated to each side of the purified PCR products following the procedure described by Meyer *et al.*, 2008. This procedure ensured that no amplification bias was introduced in contrast to procedures where barcodes are directly linked to PCR primers during the initial amplification (Berry *et al.,* 2011). Each barcode sequence was taken from a recommended barcode list described by Meyer *et al.*, 2008, and differed in at least two nucleotides from all other used barcode sequences. All barcoded PCR products were fluorometrically quantified using PicoGreen (Invitrogen, Paisley, UK) and pooled in an equimolar ratio. To avoid problems during pyrosequenicng due to simultaneous flashing of the same incorporated nucleotides when sequencing highly similar sequences (e.g., PCR products), we combined the archaeal *amoA* amplicons with PCR products from other genes for pyrosequencing (bacterial *amoA*, *nxrB* from *Nitrospira* spp., *nxrB* from *Nitrobacter* spp., data not reported in this study) as recommended previously (Kip *et al.*, 2011). Sequencing was performed on the GS FLX Titanium platform (454 Life Sciences, Branford, CT, USA) at the Norwegian High-Throughput Sequencing Centre at the University of Oslo.

SI Tables

Table S1. Highest nucleic acid sequence identity between representing sequences of the major archaeal *amoA* clusters. Presented identities were not corrected by substitution models.

	Highest sequence identity between different *amoA* clusters			
Cluster	*N'sphaera* sister	*Nitrososphaera*	*Nitrosocaldus*	*Nitrosotalea*
Nitrososphaera	83.7			
Nitrosocaldus	74.7	76.5		
Nitrosotalea	74.9	76.3	73.7	
Nitrosopumilus	78.0	78.7	74.5	83.8

Table S2. Soil samples and determined soil parameters.

Geographic location	Sample	Soil parameters					
		Latitude	Longitude	Soil pH	Total nitrogen (% dry weight)	Total org. carbon (% dry weight)	C/N ratio
Namibia [a]	dry woodland #06	18° 16' 05'' S	19° 15' 56'' E	4.60	0.042	0.565	15.7
	dry woodland #11	18° 18' 27'' S	19° 15' 46'' E	5.80	0.047	0.452	11.2
	dry woodland #16	18° 17' 14'' S	19° 15' 32'' E	6.75	0.082	0.829	11.8
	arable soil #04	18° 16' 01'' S	19° 15' 57'' E	5.84	0.034	0.305	10.5
	arable soil #08	18° 19' 10'' S	19° 18' 39'' E	6.23	0.045	0.483	12.5
	arable soil #14	18° 18' 28'' S	19° 16' 05'' E	6.17	0.038	0.392	12.0
	fallow soil #01	18° 17' 15'' S	19° 17' 28'' E	6.36	0.040	0.420	12.3
	fallow soil #10	18° 19' 11'' S	19° 18' 46'' E	6.56	0.037	0.422	13.3
	fallow soil #17	18° 19' 11'' S	19° 19' 16'' E	5.73	0.048	0.549	13.3
	fallow soil #23	18° 19' 41'' S	19° 18' 31'' E	5.95	0.028	0.290	12.1
Costa Rica	rain forest	08° 42' 16'' N	83° 12' 15'' W	4.63	0.540	7.510	16.2
	arable soil	08° 42' 03'' N	83° 12' 06'' W	4.99	0.140	1.660	13.8
Austria	riparian forest	47° 51' 09'' N	15° 03' 50'' E	7.05	0.408	3.742	10.7
	spruce forest	47° 51' 07'' N	15° 03' 52'' E	4.36	0.570	6.330	13.0
	arable soil	48° 35' 59'' N	15° 10' 24'' E	7.26	0.192	1.610	9.8
Greenland	tundra soil	69° 15' 06'' N	53° 29' 21'' W	5.09	0.200	3.980	23.2

[a] Soil samples are part of a larger biodiversity screening in Namibia (www.biota-africa.org) and therefore not consecutively numbered.

Table S3. Sequencing results and number of observed and estimated OTUs at the species level (85% *amoA* identity).

		Forward analysis / reverse analysis						
Geographic location	Sample	Number of high quality 454 reads	Good's coverage [a]	Number of observed OTUs	Chao1 richness estimator [b]	Ace richness estimator [b]	Shannon diversity index [b]	Simpson diversity index [b]
Namibia	dry woodland #06	3.153 / 2.627	0.997 / 0.998	44 / 38	52 / 42	53 / 42	1.4 / 2.0	0.45 / 0.24
	dry woodland #11	5.499 / 7.918	0.999 / 0.999	46 / 49	49 / 55	49 / 58	2.2 / 2.3	0.18 / 0.16
	dry woodland #16	3.866 / 2.623	0.999 / 0.998	35 / 33	37 / 34	38 / 35	0.8 / 1.4	0.73 / 0.43
	arable soil #04	5.770 / 4.773	0.999 / 0.998	51 / 43	65 / 46	61 / 47	1.6 / 2.0	0.39 / 0.22
	arable soil #08	4.478 / 4.581	0.998 / 0.997	52 / 53	57 / 83	59 / 73	2.5 / 2.5	0.12 / 0.13
	arable soil #14	1.330 / 1.535	0.992 / 0.997	35 / 40	50 / 43	58 / 43	2.2 / 2.5	0.19 / 0.13
	fallow soil #01	4.098 / 4.120	0.998 / 0.999	43 / 41	50 / 43	52 / 45	2.1 / 2.0	0.19 / 0.21
	fallow soil #10	7.957 / 7.534	0.999 / 0.999	46 / 47	50 / 56	52 / 56	2.2 / 2.4	0.19 / 0.13
	fallow soil #17	5.718 / 6.725	0.998 / 0.999	53 / 54	61 / 62	63 / 63	2.2 / 2.5	0.23 / 0.13
	fallow soil #23	1.953 / 1.590	0.995 / 0.992	39 / 41	48 / 54	55 / 70	2.2 / 2.3	0.19 / 0.17
Costa Rica	rain forest	2.006 / 1.985	0.997 / 0.998	9 / 8	14 / 10	21 / 16	0.04 / 0.2	0.99 / 0.93
	arable soil	2.402 / 2.344	0.998 / 0.998	10 / 15	15 / 16	31 / 26	0.1 / 0.9	0.96 / 0.50
Austria	riparian forest	1.751 / 1.791	0.996 / 0.998	27 / 18	38 / 19	38 / 21	1.6 / 1.7	0.31 / 0.26
	spruce forest	414 / 537	0.993 / 0.998	10 / 11	13 / 11	14 / 11	1.2 / 1.3	0.43 / 0.42
	arable soil	2.971 / 1.534	0.998 / 1.000	27 / 15	38 / 15	33 / 15	1.5 / 1.4	0.32 / 0.40
Greenland	tundra soil	1.870 / 2.606	0.998 / 1.000	5 / 6	8 / 6	n.p.[c] / 6	0.2 / 0.2	0.92 / 0.89

[a] based on Good, I.J. (1953) The population frequencies of species and the estimation of population parameters. Biometrika 40: 237–264.
[b] as provided by the Mothur software package (http://www.mothur.org)
[c] n.p., estimation not possible

Table S4. Correlation between beta diversity of sites (unweighted and weighted UniFrac) and measured soil characteristics as determined by Mantel's test. Determined parameters are averages based on 100 jackknifed OTU tables normalized to 1300 *amoA* sequences per soil sample.

		Correlation coefficient R^{Mantel}
Distance metric	Soil characteristic	Forward analysis / reverse analysis [a]
Unweighted UniFrac	Geographic distance [b]	0.72*** / 0.64***
Weighted Unifrac	Sum of soil parameters	0.59** / 0.60**
	pH	0.49** / 0.50**
	Total N	0.51* / 0.47*
	Organic C	0.49* / 0.49 $^{n.s.}$
	C/N ratio	0.30 $^{n.s.}$ / 0.35 $^{n.s.}$

[a] *** – p≤0.001, ** – p≤0.01, * – p≤0.05, n.s. – not significant, p>0.05
[b] Determined using partial Mantel regression keeping determined soil parameters constant.

Table S5. Number of indicator OTUs (97% *amoA* identity) in different *amoA* lineages. Only OTUs with an indicator value of 1.000 (exclusively detected under a certain tested soil characteristic) were summarized. A detailed list of individual indicator OTUs with their next relatives in public databases is given in Table S7. dw: dry weight. N: total nitrogen (% dry weight). C: total org. carbon (% dry weight).

		Phylogenetic affiliation										
Soil characteristic	Group of soil	*N'sphaera* sister subcluster 1.1	*N'sphaera* sister subcluster 2	*N'sphaera* no subcluster [c]	*N'sphaera* subcluster 1.1	*N'sphaera* subcluster 3.1	*N'sphaera* subcluster 4.1	*N'sphaera* subcluster 6.1	*N'sphaera* subcluster 7.2	*N'sphaera* subcluster 8.1	*N'sphaera* subcluster 9	*N'talea* subcluster 1.1
Geographic location	Namibia	–	–	–	4	2	–	–	–	–	1	–
	Costa Rica	–	–	–	–	–	–	–	1	–	–	1
	Austria	1	–	9	1	–	11	1	–	1	1	–
	Greenland [a]	–	–	–	–	–	–	–	–	–	–	–
pH	pH < 5.5	–	–	–	–	–	–	–	–	–	–	–
	pH=5.5-7.0 [b]	–	1	1	10	2	–	–	–	–	3	–
	pH>7.0	1	–	9	1	–	11	1	–	1	1	–
total N & org. C (%dw of soil)	N<0.08, C<0.83	–	–	–	4	2	–	–	–	–	1	–
	N=0.14-0.19, C=1.61-1.66	–	–	–	1	–	–	–	–	2	–	–
	N>0.2, C>3.7	–	–	–	–	–	–	–	–	–	–	–

[a] No statistical testing could be performed for Greenland soils because only one tundra soil was investigated.
[b] All soils from Namibia excluding Namibian dry woodland soil #06.
[c] Indicator OTUs that shared ≥85% sequences identity (species level) to a database *amoA* sequence, which did not fall into a stable subcluster, kept the cluster affiliation of the higher phylogenetic level.

Table S6 (given as separate Excel file). Representing sequences and total retrieved sequences reads of novel archaeal *amoA* OTUs at the species level cut-off of 85% identity in the individual analyzed soil samples.

Table S7 (given as separate Excel file). Indicator OTU analysis for geographic location, pH, and combined total N and organic carbon content of soils.

Table S8 (given as separate Excel file). OTU classification of *amoA* sequences at 97% identity. For each OTU the representing sequence, its affiliation, and the name and distance to the next relative in public databases is given.

SI Files

File S1. Archaeal *amoA* ARB database encompassing the consensus tree and the source alignment of sequences representing clusters at ≥97% sequence identity of all publicly available archaeal *amoA* genes by June 2010.

References

Berry, D., Ben Mahfoudh, K., Wagner, M., and Loy, A. (2011) Barcoded primers used in multiplex amplicon pyrosequencing bias amplification. *Appl Environ Microbiol*: doi: 10.1128/aem.05220-05211.

de la Torre, J.R., Walker, C.B., Ingalls, A.E., Konneke, M., and Stahl, D.A. (2008) Cultivation of a thermophilic ammonia oxidizing archaeon synthesizing crenarchaeol. *Environ Microbiol* **10**: 810–818.

Di, H.J., Cameron, K.C., Shen, J.P., Winefield, C.S., O'Callaghan, M., Bowatte, S., and He, J.Z. (2010) Ammonia-oxidizing bacteria and archaea grow under contrasting soil nitrogen conditions. *FEMS Microbiol Ecol* **72**: 386–394.

Erguder, T.H., Boon, N., Wittebolle, L., Marzorati, M., and Verstraete, W. (2009) Environmental factors shaping the ecological niches of ammonia-oxidizing archaea. *FEMS Microbiol Rev* **33**: 855–869.

Hallam, S.J., Konstantinidis, K.T., Putnam, N., Schleper, C., Watanabe, Y.-i., Sugahara, J. *et al.* (2006) Genomic analysis of the uncultivated marine crenarchaeote *Cenarchaeum symbiosum*. *Proc Natl Acad Sci USA* **103**: 18296–18301.

Hatzenpichler, R., Lebedeva, E.V., Spieck, E., Stoecker, K., Richter, A., Daims, H., and Wagner, M. (2008) A moderately thermophilic ammonia-oxidizing crenarchaeote from a hot spring. *Proc Natl Acad Sci USA* **105**: 2134–2139.

Jia, Z., and Conrad, R. (2009) Bacteria rather than archaea dominate microbial ammonia oxidation in an agricultural soil. *Environ Microbiol* **11**: 1658–1671.

Kip, N., Dutilh, B.E., Pan, Y., Bodrossy, L., Neveling, K., Kwint, M.P. *et al.* (2011) Ultra-deep pyrosequencing of *pmoA* amplicons confirms the prevalence of *Methylomonas* and *Methylocystis* in *Sphagnum* mosses from a Dutch peat bog. *Environ Microbiol Rep*: doi: 10.1111/j.1758-2229.2011.00260.x.

Könneke, M., Bernhard, A.E., de la Torre, J.R., Walker, C.B., Waterbury, J.B., and Stahl, D.A. (2005) Isolation of an autotrophic ammonia-oxidizing marine archaeon. *Nature* **437**: 543–546.

Kunin, V., Engelbrektson, A., Ochman, H., and Hugenholtz, P. (2009) Wrinkles in the rare biosphere: pyrosequencing errors can lead to artificial inflation of diversity estimates. *Environ Microbiol* **12**: 118–123.

Lauber, C.L., Hamady, M., Knight, R., and Fierer, N. (2009) Pyrosequencing-based assessment of soil pH as a predictor of soil bacterial community structure at the continental scale. *Appl Environ Microbiol* **75**: 5111–5120.

Ludwig, W., Strunk, O., Westram, R., Richter, L., Meier, H., Yadhukumar *et al.* (2004) ARB: a software environment for sequence data. *Nucl Acids Res* **32**: 1363–1371.

Martens-Habbena, W., Berube, P.M., Urakawa, H., de la Torre, J.R., and Stahl, D.A. (2009) Ammonia oxidation kinetics determine niche separation of nitrifying Archaea and Bacteria. *Nature* **461**: 976–979.

Meyer, M., Stenzel, U., and Hofreiter, M. (2008) Parallel tagged sequencing on the 454 platform. *Nat Protoc* **3**: 267–278.

Mußmann, M., Brito, I., Pitcher, A., Sinninghe Damsté, J.S., Hatzenpichler, R., Richter, A. *et al.* (2011) Thaumarchaeotes abundant in refinery nitrifying sludges express *amoA* but are not obligate autotrophic ammonia oxidizers. *Proc Natl Acad Sci USA* **108**: 16771–16776.

Pratscher, J., Dumont, M.G., and Conrad, R. (2011) Ammonia oxidation coupled to CO_2 fixation by archaea and bacteria in an agricultural soil. *Proc Natl Acad Sci USA* **108**: 4170–4175.

Tourna, M., Freitag, T.E., Nicol, G.W., and Prosser, J.I. (2008) Growth, activity and temperature responses of ammonia-oxidizing archaea and bacteria in soil microcosms. *Environ Microbiol* **10**: 1357–1364.

Tourna, M., Stieglmeier, M., Spang, A., Könneke, M., Schintlmeister, A., Urich, T. *et al.* (2011) *Nitrososphaera viennensis*, an ammonia oxidizing archaeon from soil. *Proc Natl Acad Sci USA* **108**: 8420–8425.

Verhamme, D.T., Prosser, J.I., and Nicol, G.W. (2011) Ammonia concentration determines differential growth of ammonia-oxidising archaea and bacteria in soil microcosms. *ISME J* **5**: 1067–1071.

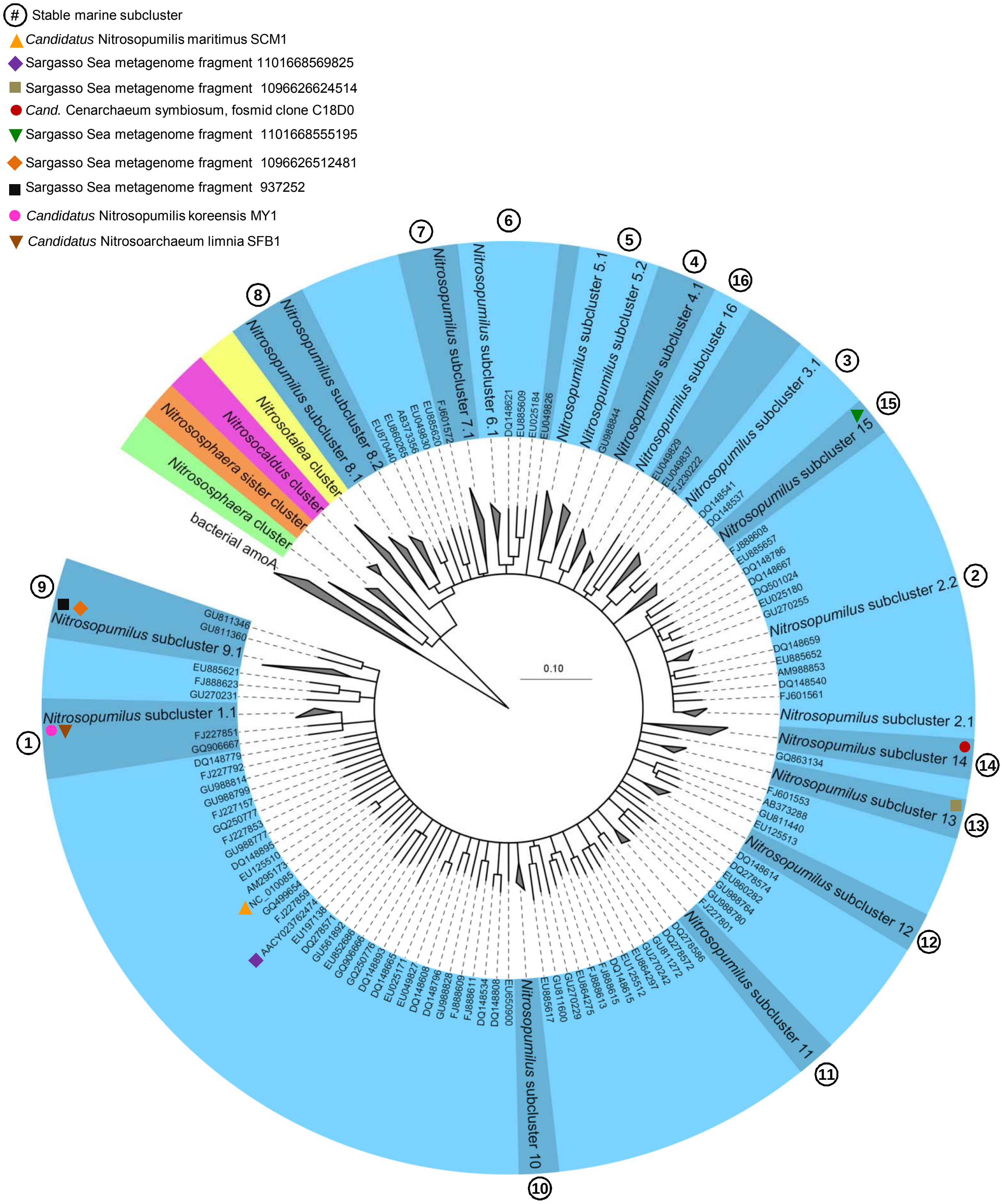

Figure S1. Consensus tree illustrating the diversification of the *Nitrosopumilus* cluster at the second and third phylogenetic level. The tree was determined using 592 unambiguously aligned positions of 735 nucleic acid sequences that evenly cover the known sequence space of archaeal *amoA* at a ≥97% sequence identity level. Numbers in circles represent the second phylogenetic level (e.g., *Nitrosopumilus* subcluster 1), whereas the third phylogenetic level is directly indicated at the tree branch (e.g., *Nitrosopumilus* subcluster 1.1); sequences that did not form stable sublineages of more than three representatives kept the affiliation of the higher phylogenetic level and are indicated by their NCBI accession number. The consensus tree and the source alignment of representing sequences can be found in File S1. The scale bar indicates 10% estimated sequence divergence.

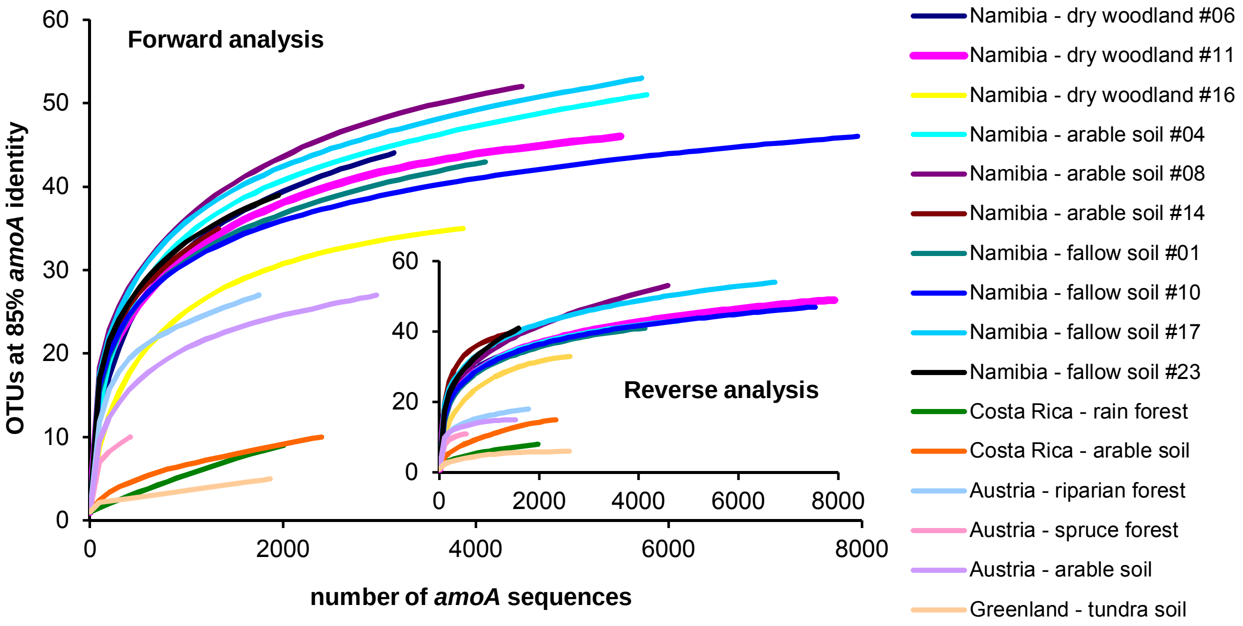

Figure S2. Rarefaction analysis of forward and reverse sequenced *amoA* at the species level cut-off of 85% *amoA* identity.

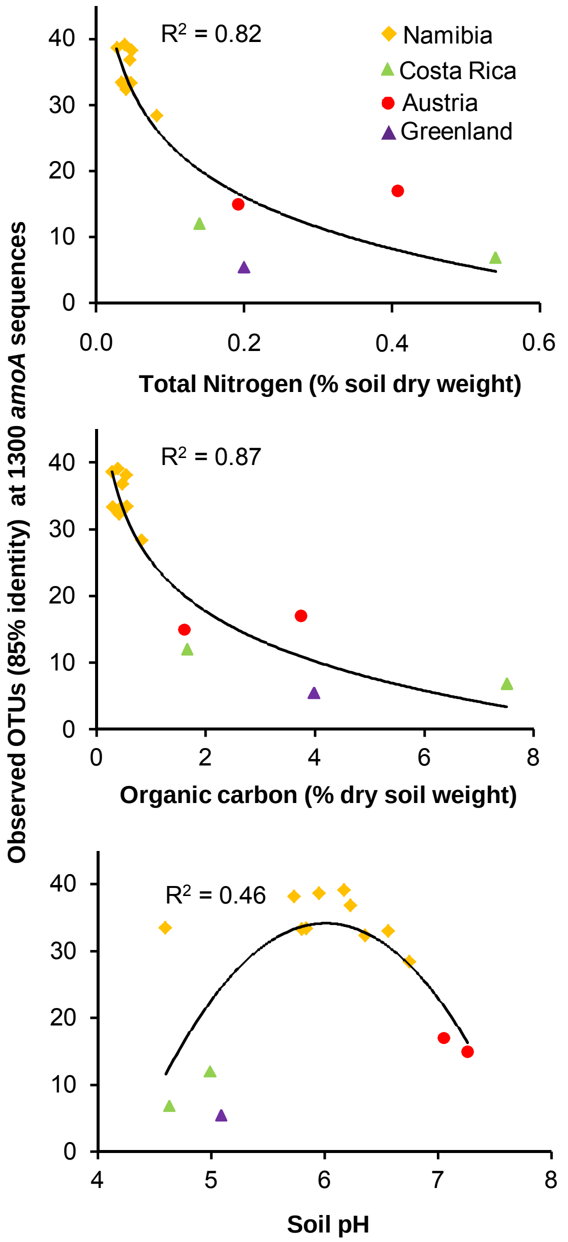

Figure S3. Correlation analysis of total nitrogen, organic carbon, and soil pH to OTU richness at the species level cut-off of 85% *amoA* identity when normalized to 1300 reads per soil and sequencing direction. The Austrian spruce forest was omitted from the analysis due to a sequence number of less than 1300 reads. The analysis of the reverse sequences is shown with all correlations having a p-value <0.05; analyses of forward sequences gave similar results (data not shown). Highest AOA species richness was observed at the lowest total nitrogen and organic carbon content, which agrees well with the cumulative recent findings that AOA are generally adapted to low ammonia concentrations and are inhibited by high loads of dissolved organic carbon (de la Torre et al., 2008; Di et al., 2010; Erguder et al., 2009; Hatzenpichler et al., 2008; Könneke et al., 2005; Martens-Habbena et al., 2009; Pratscher et al., 2011; Verhamme et al., 2011). The few detected AOA species in soils with high loads of nitrogen and organic carbon indicate the existence of ecotypes adapted also to these conditions or represent AOA that perform a mixotrophic or heterotrophic lifestyle (Hallam et al., 2006; Jia and Conrad 2009; Mußmann et al., 2011; Tourna et al., 2011). For soil pH, AOA species richness followed the general trend of microbial species richness observed in a large survey of soils (Lauber et al., 2009), with a maximum of species at slightly acidic pH (pH=6).

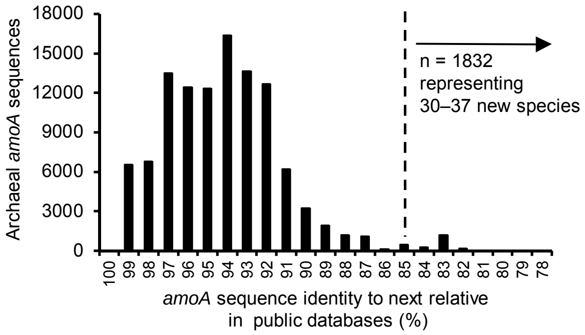

Figure S4. Abundance plot showing sequence identities of soil archaeal *amoA* retrieved by 454 pyrosequencing to next relatives in public databases. The approximate species-level threshold of 85% *amoA* sequence identity is indicated by a dotted line.

```
                                     1          11         21         31
                                     |          |          |          |
CamoA-19f                            ATGGTCTGGY TWAGACG
Nitrosopumilus maritimus SCM1        ATGGTCTGGT TAAGACGATG TACACACTAC ---
Nitrosoarchaeum koreensis MY1        ATGGTCTGGC TAAGACGATG TACTCACTAC ---
Nitrosoarchaeum limnia               ATGGTCTGGC TAAGACGATG TACTCACTAC ---
Cenarchaeum symbiosum fosmid C18D02  ATGGTCTGGC TAAGACGCTG TACCCACTAC ---
Nitrosotenuis uzonensis (unpublished) ATGGTCTGGC TTAGACGATG TACTCACTAC ---
Sargasso Sea WGS 1101668569825       ATGGTCTGGT TAAGACGATG TACTCACTAC ---
Sargasso Sea WGS 1096626624514       ATGGTCTGGT TAAGACGATG TACACACTAC ---
Sargasso Sea WGS 1101668555195       ATGGTCTGGC TTAGACGATG TACTCATTAC ---
Sargasso Sea WGS 1096626512481       ATGGTCTGGC TTAGACGATG TACTCACTAC ---
Sargasso Sea WGS 937252              ATGGTCTGGC TTAGACGATG TACTCACTAC ---
Nitrosotalea devanaterra             ATGGTCTGGC TAAGACGATG TACTCACTAC ---
Nitrosocaldus yellowstonii           ATGGTCTGGG TTAGAAGGAC TATACACTAC ---
Nitrososphaera gargensis             ATGGTCTGGC TTAGACGTAC AACGCACTAC ---
Nitrososphaera viennensis            ATGGTCTGGC TTAGACGTAC AACGCACTAC ---
Sandy soil fosmid 54d9               ATGGTCTGGC TTAGACGCAC TACACACTAT ---

                                         591        601        611        621        631        641        651
                                         |          |          |          |          |          |          |
CamoA-616r                                                       TGGACNTA YMGVTGGATG GC
Nitrosopumilus maritimus SCM1        --- GTTAGGTTGT AAATTGAATA CATGGACTTA CAGATGGATG GCCGCTTGGT CAAAGTGGGA CTAA
Nitrosoarchaeum koreensis MY1        --- ATTAGGTTGT AAGCTAAACA CTTGGACATA CAGATGGATG GCCGCTTGGT CAAAGTGGGA CTAA
Nitrosoarchaeum limnia               --- ATTAGGTTGT AAATTAAACA CTTGGACATA CAGATGGATG GCCGCTTGGT CAAAGTGGGA CTAA
Cenarchaeum symbiosum fosmid C18D02  --- CTTGGGTGTC AAGCTCAATA CGTGGACGTA TCGGTGGATG GCCGCTTGGT CAAAGTGGGA CTAG
Nitrosotenuis uzonensis (unpublished) --- GTTGGGATGC AAGCTAAATA CGTGGACGTA TAGATGGATG GCAGCTTGGT CCAAGTGGGA CTAA
Sargasso Sea WGS 1101668569825       --- ATTAGGTTGT AAGCTAAACA CTTGGACTTA CAGATGGATG GCCGCTTGGT CAAAGTGGGA CTAA
Sargasso Sea WGS 1096626624514       --- ACTAGGTTGT AAATTGAACA CTTGGACATA CAGATGGATG GCCGCATGGT CAAAGTGGGA CTAA
Sargasso Sea WGS 1101668555195       --- GTTAGGTTGT AAGCTAACTA CGTGGACATA TAGATGGATG GCTGCTTGGT CAAAGTGGGA CTAA
Sargasso Sea WGS 1096626512481       --- TTTAGGTTGT AAACTTACAA CTTGGACATA CAGATGGATG GCCGCTTGGT CAAAGTGGGA TTAG
Sargasso Sea WGS 937252              --- CTTAGGTTGT AAACTAACAA CGTGGACATA CAGATGGATG GCCGCTTGGT CAAAGTGGGA TTAG
Nitrosotalea devanaterra             --- ATTGGGTTGC AAATTAACAA CATGGACATA CAGATGGATG GCAGCCTGGT CTAAGTGGGA CTAA
Nitrosocaldus yellowstonii           --- CTTAGGAGCA AAGTTGAATA CATGGACATA CAGATGGGCT GCAGCTTGGA GCAAGTGGGA CTAG
Nitrososphaera gargensis             --- ATTGGGTGCG AAGCTTAACA CGTGGACATA CAGATGGATG GCCGCGTGGA GCAAGTGGGA CTAA
Nitrososphaera viennensis            --- GTTGGGTGCG AAACTTAACA CGTGGACATA CAGATGGATG GCCGCGTGGA GCAAGTGGGA CTAA
Sandy soil fosmid 54d9               --- ATTGGGCGCA AAACTAAATA CTTGGACATA CAGATGGATG GCCGCCTGGT CTAAGTGGGA TTAA
```

Figure S5. *In silico* specificity analysis of the archaeal *amoA* primers used in this study against all archaeal *amoA* sequences covering the primer target regions. The primer regions of *Candidatus* Nitrosotalea devanaterra, *Candidatus* Nitrosocaldus yellowstonii, and *Candidatus* Nitrosotenuis uzonensis (affiliated to *Nitrosopumilus* subcluster 5.1) represent partly unpublished data and were kindly provided by Graeme Nicol (Institute of Biological and Environmental Sciences, University of Aberdeen), José de la Torre (Department of Biology, San Francisco State University) and Roland Hatzenpichler and Susanne Haider (Department. of Microbial Ecology, University of Vienna), respectively.

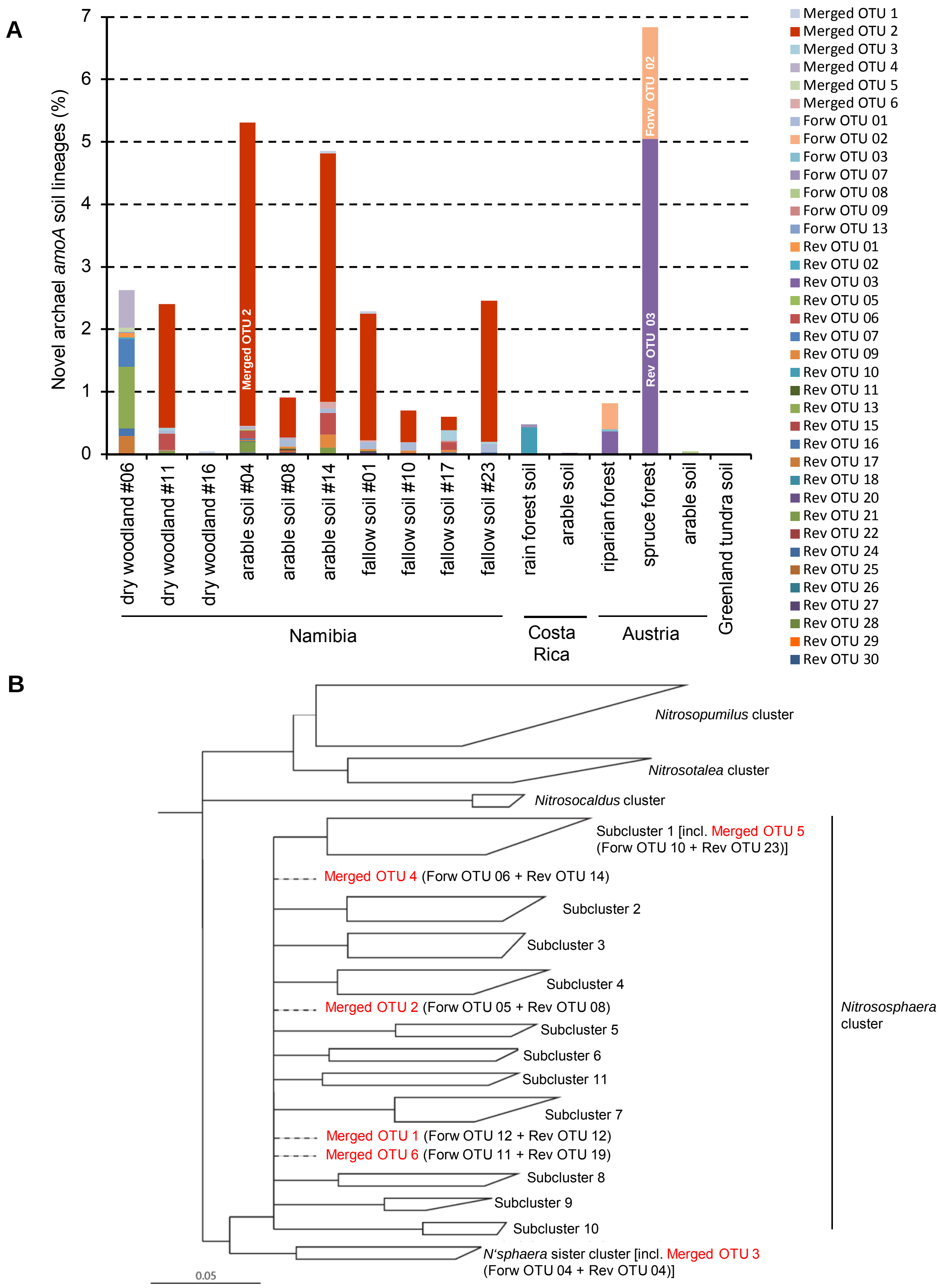

Figure S6. Analysis of novel *amoA* that shared less than 85% sequence identity to deposited sequences in public databases. (A) Abundance of novel OTUs at the species level cut-off of 85% *amoA* identity in the respective samples. Representing sequences of forward and reverse OTUs that overlapped by more than 260 nt and shared at least 97% sequence similarity (within the 454 sequencing error range, Kunin *et al.*, 2009) were merged to represent one OTU. A detailed list of novel OTUs including their representing sequence is given in Table S6. (B) Phylogenetic position of selected novel *amoA* using representing sequences of all merged OTUs. The phylogenetic position of representing sequences of novel *amoA* OTUs within the *amoA* consensus tree was deduced by two independent inference methods: (i) the interactive parsimony tool within the ARB software package (Ludwig et al., 2004) and (ii) and a distance matrix method (neighbor joining tree based on a Jukes-Cantor corrected distance matrix). Thereafter, novel *amoA* OTU representatives were added manually to the archaeal *amoA* consensus tree (Fig. 1) without changing the overall tree topology (as indicated by the dotted branches of uniform length of the added sequences).

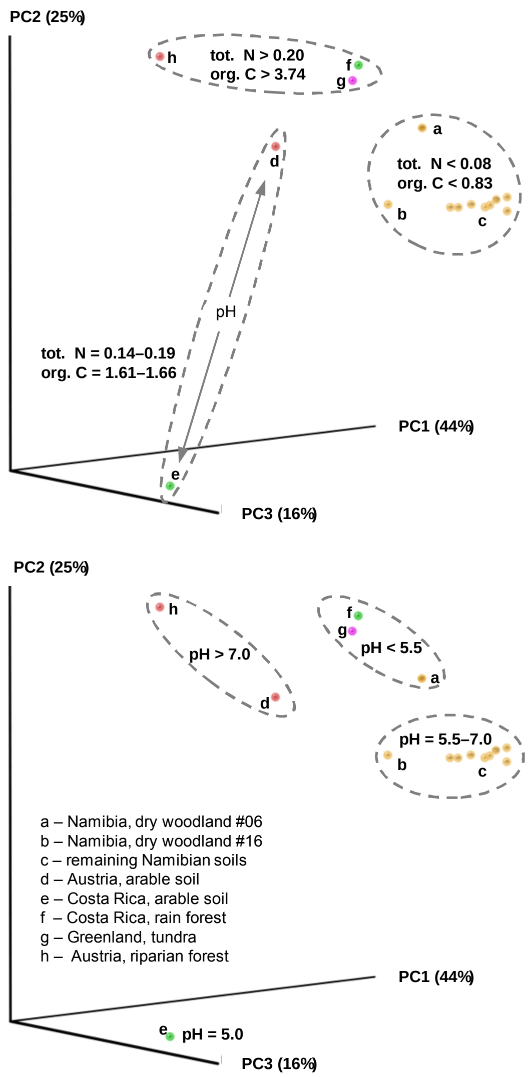

Figure 7. Principal component analysis based on OTU abundance (jackknifed weighted UniFrac) and separating soils according to their combined total nitrogen/organic carbon content or to soil pH. For this analysis, observed *amoA* OTUs at 97% sequence identity were used (representing the highest possible phylogenetic resolution) and normalized to 1300 reads per soil and sequencing direction. The Austrian spruce forest soil was omitted from the analysis due to a sequence number of less than 1300 reads. Analysis of the forward sequences is shown; analysis of reverse sequences gave similar results (data not shown).
